# Tuning the optical response of a dimer nanoantenna using plasmonic nanoring loads

**DOI:** 10.1038/srep09813

**Published:** 2015-05-11

**Authors:** Anastasios H. Panaretos, Yu A. Yuwen, Douglas H. Werner, Theresa S. Mayer

**Affiliations:** 1 Department of Electrical Engineering, The Pennsylvania State University, University Park, Pennsylvania 16802, United States

## Abstract

The optical properties of a dimer type nanoantenna loaded with a plasmonic nanoring are investigated through numerical simulations and measurements of fabricated prototypes. It is demonstrated that by judiciously choosing the nanoring geometry it is possible to engineer its electromagnetic properties and thus devise an effective wavelength dependent nanoswitch. The latter provides a mechanism for controlling the coupling between the dimer particles, and in particular to establish a pair of coupled/de-coupled states for the total structure, that effectively results in its dual mode response. Using electron beam lithography the targeted structure has been accurately fabricated and the desired dual mode response of the nanoantenna was experimentally verified. The response of the fabricated structure is further analyzed numerically. This permits the visualization of the electromagnetic fields and polarization surface charge distributions when the structure is at resonance. In this way the switching properties of the plasmonic nanoring are revealed. The documented analysis illustrates the inherent tuning capabilities that plasmonic nanorings offer, and furthermore paves the way towards a practical implementation of tunable optical nanoantennas. Additionally, our analysis through an effective medium approach introduces the nanoring as a compact and efficient solution for realizing nanoscale circuits.

The theoretical development of optical plasmonic nanoantennas has greatly benefited by the fact that their principles of operation to a large extent resemble those of their radio frequency and microwave counterparts 1,2,3,4,5,6,7,8,9,10. As a consequence there is a plethora of analysis techniques and design methodologies that have been and could be adopted towards the development of efficient optical nanoantennas. Therefore, it is not surprising that so far researchers have studied and developed optical nanodipole, bowtie, and Yagi-Uda type nanoantennas. However, despite the large number of possible nanoantenna designs, their practicality and realization are subjected to the limitations that nanofabrication techniques impose. In other words, the more advanced and accurate nanofabrication processes become, the more feasible it is to realize nanoantenna configurations with a higher degree of sophistication, which will effectively permit the designer to achieve greater functionality out of a device.

The radiation properties of a nanoantenna are primarily determined by its shape. Usually the design begins by defining first a baseline radiation element, followed by an appropriate modification so that certain specifications or wider functionality can be achieved. One way to realize this is by introducing some type of tuning mechanism, which essentially requires that the baseline structure be loaded with a device that permits manipulation of the nanoantenna’s response as desired. In the case of a nanodipole it has been demonstrated that its impedance, and thus its radiation properties, can be tuned by filling the loading volume defined in-between its two arms with different materials 11,12. This type of tuning approach has been experimentally demonstrated in 13,14 and 15. In the latter paper the response of a nanodimer comprised of circular disks is tuned by loading the volume defined between the two disks with different material combinations. Similarly, in the first two papers an effective loading scheme is devised by removing small bits of material from around the center of a nanobar. In both cases, the structure modification creates an effect equivalent to that of an electrically small circuit that loads and tunes the impedance response of a nanoantenna. An alternative type of tuning mechanism has been documented in 16, where the loading volume of a plasmonic nanodipole was filled with photoconductive material. In this case the material operates as a nanoswitch which enables either the coupling or decoupling of the two nanodipole arms. From the preceding analysis it becomes evident that one of the great challenges for the realization of effective tuning schemes at the nanoscale is the development and fabrication of nanodevices than can effectively load and tune a nanoantenna configuration.

In this paper through experiments and numerical simulations we investigate the feasibility of tuning the optical response of a nanodimer using plasmonic nanorings. It has been well-documented that the resonance properties of plasmonic nanodimers are primarily determined by the gap distance between their two constituent particles 17,18,19. Herein we demonstrate that a plasmonic nanoring can function as an effective nanoswitch that enables the dual mode operation of the loaded nanodimer structure. Plasmonic nanorings are ideal candidates for this type of functionality since their electromagnetic properties are inherently characterized by a switching response due to the two distinct hybridized dipole moments that this geometry can support 20,21,22,23,24. It should be noted here that this manuscript can be considered as a companion to our previously published work in 25. In that paper it was theoretically demonstrated how the response of a nanodipole can be custom engineered using plasmonic core-shell particles. In the current manuscript a planar version of that nanoantenna configuration is studied where the cylindrical nanodipole is substituted by a circular disk dimer, while the core-shell load is represented by a nanoring. Our analysis reveals that if the geometrical characteristics of the nanoring are carefully chosen then its two dipole moments can be spectrally arranged so that the loaded dimer exhibits a dual mode operation. The radiation properties of these modes correspond to the states where the two particles of the dimer radiate either independently or collectively.

## Results

### Geometrical considerations for choosing the base nanoantenna structure

**Figure 1:**
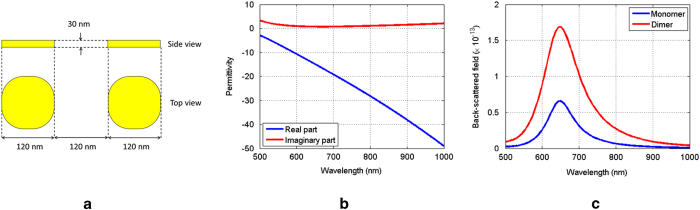
(**a**) Baseline geometry. (**b**) Measured dielectric properties of gold. (**c**) Backscattered response of the baseline dimer and one of the particles (monomers) that comprise the dimer.

At the first stage of this study it was required to determine through numerical simulations the geometrical characteristics of a nanodevice that would facilitate demonstration of the switching functionality under investigation. This stage was of paramount importance because the final design should correspond to a realizable structure taking into consideration the constraints of available nano-fabrication facilities. After extensive numerical experimentation it was decided that our baseline structure should be the nanodimer shown in Fig. 1(a). We define the baseline structure as one whose optical response can be tuned to enable its dual mode operation. The dimer is comprised by two equal in size gold parallelepipeds with a height of 30 nm. The cross-section of each parallelepiped is 120 nm–by–120 nm, while its corners have been blended at a radius of 50 nm. Also, the distance in between the two particles is equal to 120 nm. In order to be consistent with the actual measurement set-up, the structure is modeled to reside on an infinite glass substrate with permittivity equal to 2.09. The dielectric properties of gold, shown in Fig. 1(b), are defined according to measurements performed on fabricated gold samples.

Now, given that the separation distance between the two disks is comparable to their radius, it is expected that upon illumination by a plane wave, there will be insignificant coupling between the two elements. It should be emphasized here that in this study we are interested in “structure modes” as opposed to individual particle resonances. Therefore, unless it is stated otherwise, the structures under study are illuminated by a plane wave polarized parallel to their center-to-center vector. This is the proper polarization required in order to excite the structure modes which are of interest in this work. Due to the minute coupling between the two particles comprising the dimer, the scattering signature of the compound structure corresponds to the superposition of the scattering response from the two individual disks, and therefore they exhibit the same resonance wavelength as shown in Fig. 1(c).

Given that in this particular case the size and spatial arrangement of the two disks is fixed it is expected that the structure will need to be appropriately loaded in order to tune its optical response, or equivalently it should be structurally modified. In this case we adopt a loading scheme analogous to that of a radio frequency (RF) wire dipole antenna 26,27, which suggests treating the space in between the two disks as the nanodimer’s “loading volume”. It should be emphasized that in principle the loading volume of a radiating element is electrically small which obviously is not the case for the baseline nanoantenna structure. However, as it will be demonstrated later in our analysis, even in this extreme case electrical responses that resemble those of a tuning circuit load can still be realized, which is advantageous from a nanofabrication perspective.

As mentioned previously the objective is to devise a loading particle that permits the dual-mode operation of the nanodimer. This can be realized if we choose a load for our structure whose electromagnetic properties resemble those of a tunable wavelength dependent nanoswitch. Essentially, the nanoswitch regulates the coupling mechanism between the particles comprising the dimer thereby allowing the desired optical response to be achieved. The engineering of a custom wavelength dependent response for the nanoswitch is primarily determined by two design parameters: its material properties and its geometrical characteristics. Given that, material customization is difficult to achieve at the nanoscale, the proposed design solely relies on customizing the geometry of the nanoswitch. To this end, nanorings were selected as the most promising candidate geometry in order to enable the desired switching capability.

There are two main advantages offered by the nanorings: first, since structure modes are strongly dependent on the coupling mechanism between the dimer and the load particle, the spacing between them is a critical parameter in the design. This by itself imposes great fabrication limitations since it is not that straightforward to maintain nano-meter scale accuracy during the fabrication process. A nanoring however allows the interparticle distance to be maintained, by keeping its outer radius constant (or within some acceptable tolerance), while its electromagnetic properties can be tuned by modifying its more forgiving inner radius. Second, although the electromagnetic properties of plasmonic nanorings are traditionally explained in terms of mode hybridization models, it can be shown that nanorings may be considered as homogeneous disks characterized by effective dielectric properties similar to those of a medium governed by the Maxwell-Garnett mixing rule. In other words, by modifying the geometrical characteristics of the nanoring we essentially create disks characterized by effective material properties that could not otherwise be easily achieved at optical wavelengths.

**Figure 2:**
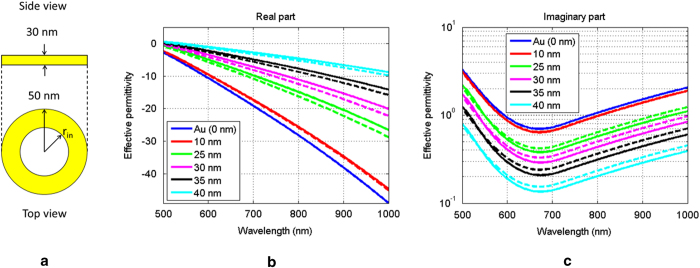
(**a**) Nanoring geometry. (**b**) Real part and (**c**) imaginary part of the effective dielectric properties of the nanoring. Solid lines correspond to numerically computed values using (1). Dashed lines correspond to values computed using (2).

In order to demonstrate the previous statement let us consider the ring geometry shown in Fig. 2(a). Its outer radius is equal to 50 nm while for its inner radius *r*_*in*_ we examine the following values: 10 nm, 25 nm, 30 nm, 35 nm, and 40 nm. The height of the nanoring is set equal to 30 nm. The structure is immersed in free space, with the ring comprised of gold and the void volume filled with free space. The nanoring is excited by a plane wave polarized parallel to its diameter. For each simulation performed, which corresponds to a different value of the inner ring radius, the following quantity was computed:
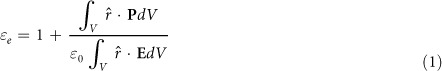


In the preceding formula 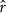 and 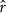 are respectively the polarization and electric field intensity components that are parallel to the incident plane wave’s polarization vector 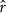. Also, 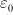 is the permittivity of free space while the integration is performed over the volume of the nanoring. This volume averaging based formula yields a rough albeit indicative approximation of the nanoring’s effective dielectric properties. The corresponding results for the calculated effective permittivity are illustrated by the solid lines in Fig. 2(b,c). In the same figures, the dashed lines represent the effective permittivity as predicted by the Maxwell-Garnett mixing rule for a mixture comprised of infinite-in-length circular cylinders, or


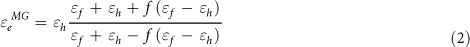


where in this case the permittivity of the host material 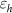 is gold (ring constitution), and the permittivity of the filler material 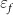 is free space (void constitution). The *area* fraction in the preceding formula is defined as 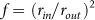.

First, it can be seen that as the inner radius increases the effective dielectric properties of the ring red-shift with respect to the dielectric properties of gold. Second, there is good agreement between the Maxwell-Garnett model and the calculated effective permittivity values as given by (1). In other words, there is clear indication that the nanoring can be characterized by its internal nano-mixture properties, where the material of the void (free-space) is diluted in the material of the host (gold). The effective material is Lorentzian in nature and its properties are solely dependent on the value of the area fraction, given that the constituents of the mixture are fixed. It should be noted that the observed discrepancies are attributed to the fact that (2) assumes infinite in length circular cross section cylindrical fillers, while in this case the height of the mixture is finite and equal to 30 nm. Another reason for the observed discrepancies is that (2) is applicable when the mixture’s area fraction is low, and additionally when the size of each cylinder is electrically small. It should be mentioned here that given the direct relationship between the admittance and material properties of a structure, the nanoring under study corresponds to a device characterized by tunable admittance properties where the tuning parameter is solely dependent on the geometrical characteristics of the structure. As a result it is expected that by changing the value of the inner radius, the ring’s effective material properties will be altered, and subsequently this will tune accordingly the way the ring couples with the dimer.

**Figure 3:**
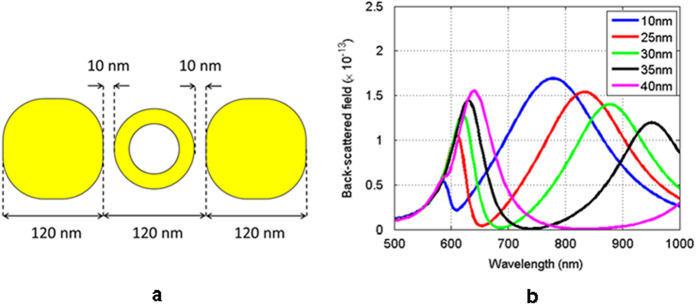
(**a**) Nanoring loaded baseline geometry. (**b**) Backscatter response for different values of the inner radius of the nanoring.

Having established a qualitative framework to explain the electromagnetic response of a plasmonic nanoring, we proceed with the investigation for a suitable nanoring geometry that will enable the desired dual-mode response of the baseline dimer. Fig. 3(a) shows the corresponding geometry and, as explained previously, it is excited by a plane wave polarized parallel to the vector defined by the dimer’s center-to-center distance. The backscattered response corresponding to the five different values of the inner radius is shown in Fig. 3(b).

From Fig. 3(b) it can be clearly seen that for the spectrum of interest the scattering response of the trimer exhibits two resonance peaks. The features of these peaks vary as a function of the nanoring’s inner radius. In particular, it can be seen that the width of the long wavelength resonance decreases and the width of the short wavelength resonance increases. This behavior can be justified as follows: the short wavelength resonance corresponds to the scattering resonance of each half of the dimer. As the ring’s inner radius becomes smaller the coupling between the left and right particle decreases. In the limit where the inner radius is equal to the outer radius the ring vanishes, and consequently the width of the short wavelength resonance is maximized, which can be observed in the dimer scattering simulation results shown in Fig. 1(c). In contrast, the long wavelength resonance corresponds to the case where the three particles scatter as a longer effective homogeneous particle. Now, as the inner radius of the ring increases the effective volume of the structure decreases, and therefore the bandwidth of the resonance increases. More insight into the coupling mechanism between the three particles will be given in a later section. In conclusion, from the previous parametric study it is evident that for the given structure an inner radius between 25 nm and 35 nm is required in order to provide a clear demonstration of the coupling/decoupling phenomenon for the trimer, thus exhibiting its dual mode operation.

### Experimental setup and measurements

**Figure 4:**
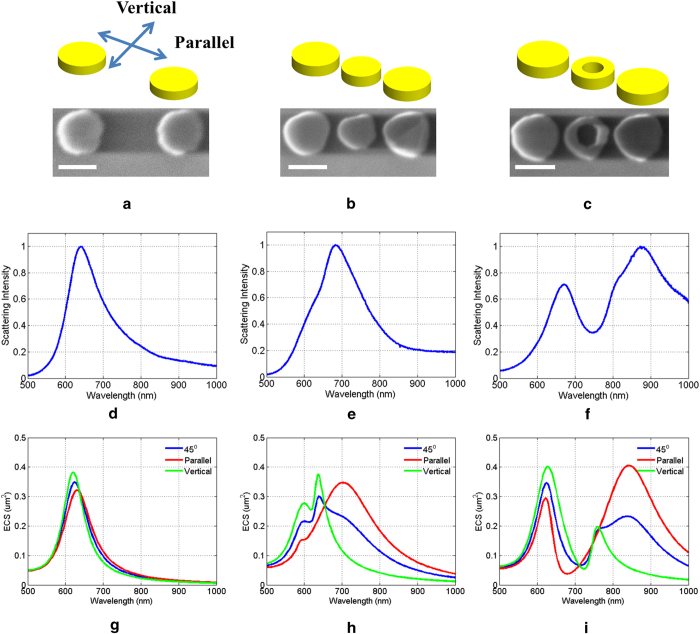
Measured and simulated optical response of the nanodimer for the three different loading scenarios. (**a**) SEM image of dimer with no load, (**b**) SEM image of dimer with solid circular disk load, and (**c**) SEM image of dimer with nanoring load. The white scale bar corresponds to a 100 nm length. Measured normalized scattering intensity of the nanodimer with (**d**) no load, (**e**) solid circular disk load, and (**f**) nanoring load. Simulated extinction cross section of the nanodimer with (**g**) no load, (**h**) solid circular disk load, and (**i**) nanoring load.

Fabrication of the nanoring with the desired dimensions as described in the previous section (25 nm inner radius and 50 nm outer radius) was a very challenging task. The details of this process are described in the Device Fabrication subsection of the Methods section. Now, the objective of this study was to systematically demonstrate the effects of the loading particle in the baseline dimer’s optical response. For this reason three different structures were fabricated, corresponding to three different loading scenarios, and subsequently their optical response was measured. In particular, first we measured the optical responses of the unloaded dimer. Following, we examined the cases where the dimer is loaded by a solid circular disk, and then finally by a nanoring. The SEM images of these fabricated nanostructures are shown in Fig. 4(a–c).

The longest diameter of the two particles comprising the dimer shown in Fig. 4(a) is 116 nm which is slightly shorter than the desired length, which is 120 nm. Moreover, for the case of the solid disc load shown in Fig. 4(b), the length of the gap between the solid load and the dimer particles is 1 nm longer than the targeted length, which is equal to 20 nm. Finally, for the nanoring loaded dimer shown in Fig. 4(c), one discrepancy between the fabricated and the desired geometry concerns the length of the two gaps defined between the three particles. In particular, with respect to the image in Fig. 4(c), the left and right gap lengths are equal to 14 nm and 16 nm, respectively, while the desired length would be 10 nm. Additionally, the left and right particles of the trimer have slightly different diameters: the diameter of the left one is 121 nm, while that of the right one is 123 nm. Finally, the length of the fabricated nanoring’s outer diameter is 10 nm longer than the desired value, while the diameter of the inner circle is equal to approximately 61 nm. All of the aforementioned geometry discrepancies were taken into account for the development of the numerical models presented in the next section.

A series of far-field scattering measurements were performed in order to characterize the optical response of the three fabricated nanostructures, as described in the Optical characterization subsection of the Methods section. The optical response of the three nanoantennas, measured under identical conditions, is shown in Fig. 4(d–f). The dimer structure exhibits only one resonance peak around 630 nm. The trimer configuration corresponding to the dimer loaded with a solid circular disk, as expected exhibits a red-shifted resonance compared to the response of the dimer, while the width of this resonance is slightly wider. The resulting red-shift is a direct consequence of the coupling that is introduced between the solid load and the dimer. Finally, in Fig. 4(f) it can be clearly seen that when the dimer is loaded by the nanoring the compound structure exhibits two resonances around 650 nm and 870 nm respectively. This behavior is in accordance with the numerically predicted results reported in the previous section, where the response of the trimer was studied as a function of the nanoring’s inner radial length.

### Numerical validation

**Figure 5:**
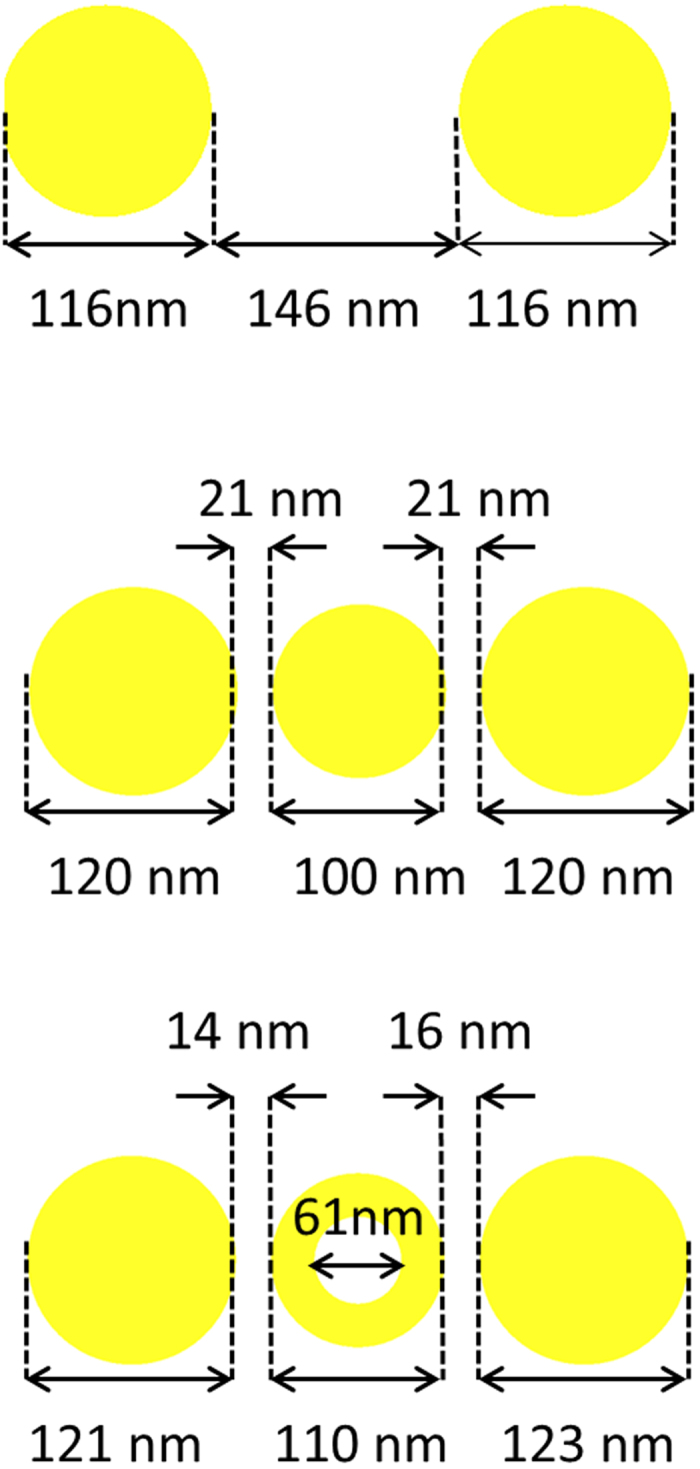
Geometrical characteristics of the numerically simulated nanoantenna with (**a**) no load; (**b**) solid load; (**c**) nanoring load.

The experimentally obtained optical response of the three fabricated nanostructures was validated through a series of numerical simulations. This not only allows the accuracy of the experimental procedure to be assessed, but also helps to reveal the underlying physics behind the coupling mechanism that enables the switching functionality of the nanoring, and thus the dual mode operation of the loaded dimer. In order to achieve computationally meaningful and realistic results, the geometrical characteristics of the numerically modeled structures were chosen to be as close as possible to those of the actual fabricated prototypes. These characteristics were described in the previous section, but for clarity and convenience we further summarize them in Fig. 5. All numerical simulations were performed using the Comsol Multiphysics full-wave electromagnetics solver. As mentioned previously, this effort is focused on investigating the antenna mode of the nanostructures under study.

This mode is typically excited when the nanoantenna is illuminated by a plane wave polarized parallel to its long dimension. However, in order to obtain a more comprehensive understanding of the structures’ scattering response, we simulated two additional cases for the polarization of the plane wave excitation. In summary, the three scenarios examined correspond to linearly polarized plane waves, propagating in a direction perpendicular to the plane containing the nanostructure. The polarization of the three incident plane waves is 0°, 45°, and 90° with respect to the long dimension of the structure. The first case, denoted as “parallel polarization,” is responsible for the antenna mode excitation; the third case, denoted as “vertical polarization,” primarily results in the individual excitation of each particle. Finally, the 45° polarized plane wave closely resembles the “un-polarized incident light” scattering scenario, and naturally it results in the average scattering response of the structure, when the latter is stimulated simultaneously by both a parallel and a vertical excitation.

The lowest row of Fig. 4 summarizes the extinction cross section predictions for the three nanostructures subject to the aforementioned illumination scenarios. A cursory examination of the responses shown in Fig. 4(g) reveals that the coupling between the two particles is minute since its optical response is nearly independent of the incident plane wave’s polarization. As a matter of fact a progressive, albeit slight, red-shift is observed as the transition is made from the vertical to the parallel excitation. Despite this minor variation, the three responses primarily correspond to the superposition of the extinction cross-section of the two particles. Finally, it should be emphasized that very good agreement is observed between the numerically predicted response and the experimental measurements, as shown in Fig. 4(d). Note here that the documented measurements have been obtained after exciting the nanostructures with unpolarized light. Therefore, the obtained results correspond to the superposition of all linear polarizations at angles from 0° to 180° (not 360° due to symmetry). Therefore, the numerical predictions and the measurements should be compared in an average sense rather than by making a direct one-to-one comparison. This last remark holds for all three of the scattering scenarios examined here. Now, the optical response of the dimer drastically changes when it is loaded by the solid circular disc, as is evident from Fig. 4(h). In particular, a pronounced red-shift is observed when the trimer is excited by the parallel polarized plane wave. Evidently, the three particles couple constructively and thus the trimer scatters as an effective nanobar. The effective length of the latter is longer than the size of the individual particle, and thus the supported antenna mode occurs at a longer wavelength. In contrast, the vertically excited structure does not support any structure mode, and the observed resonances correspond to the individual plasmon resonances of the trimer’s component particles. Finally, the optical response that corresponds to the 45° polarization clearly exhibits the combined characteristics of the obtained optical response due to the parallel and the vertical excitations. Again, very good agreement is observed between the numerically predicted response and the experimentally obtained measurements, which are shown in Fig. 4(e).

Before proceeding with the analysis of the nanoring loaded dimer’s extinction response, it is worth mentioning that the aforementioned loading scenarios (no load and solid disc load) correspond to the two limiting scattering states that such a structure can support. In particular, the “no load” case represents the state where the dimer particles are decoupled and scatter individually. On the other hand, upon insertion of the solid disc load the “coupled state” of the particles is established and the compound structure exhibits a red-shifted resonance. Note here that the solid disc load used in this study was not chosen so that a maximum coupling effect could be achieved. Its geometrical characteristics were rather chosen so that, to a certain degree, the concept of coupling could be demonstrated. The objective now becomes the selection and implementation of a wavelength dependent switching particle, where in this case it is the nanoring, so that both of the aforementioned scattering states can be enabled. Indeed, as can be clearly seen in Fig. 4(i) for the parallel excitation, the structure exhibits two distinct resonances. Moreover, from the wavelengths at which these resonances occur, as well as from their linewidth, they can be attributed to the coupled and decoupled states of the trimer. It should be emphasized here that the nature of these two resonances should by no means be confused with the two resonances that the extinction spectrum exhibits, when the structure is vertically excited. As explained previously, these are the resonances of the individual particles, and cannot be categorized as antenna modes. Finally, the experimentally obtained spectral response for the unpolarized excitation shown in Fig. 4(f) could be considered as a superposition of the optical responses created by a collection of polarization excitations that span from parallel to vertical. This experimental response is in very good agreement with the numerically obtained response illustrated in Fig. 4(i). In what follows, we further elucidate the origin of the multi-resonance response of the nanoring loaded dimer by examining the electric and magnetic field distributions as well as the polarization charge distribution of the resonating structures.

### Electric field, magnetic field, and polarization charge distributions

**Figure 6:**
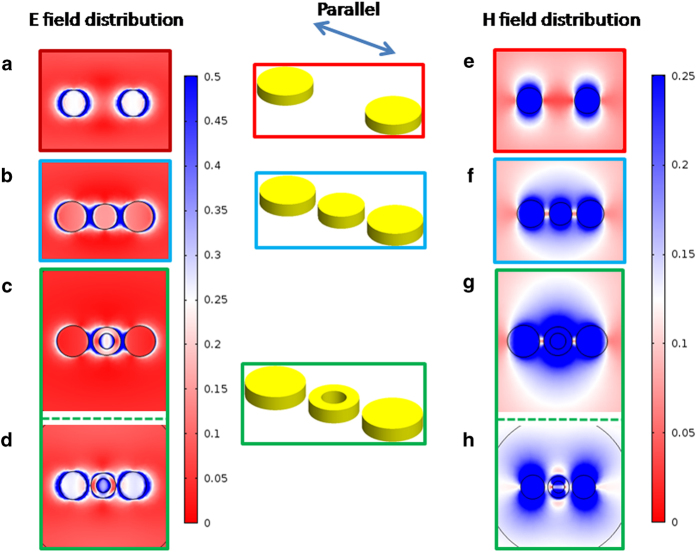
Electric and magnetic field distributions at the resonance wavelength for parallel polarization incidence. (**a**) and (**d**) No load computed at 632 nm. (**b**) and (**e**) Solid circular disk load computed at 705 nm. (**c**),(**d**) and (**f**),(**g**) Ring load computed at 845 nm and 622 nm, respectively.

**Figure 7:**
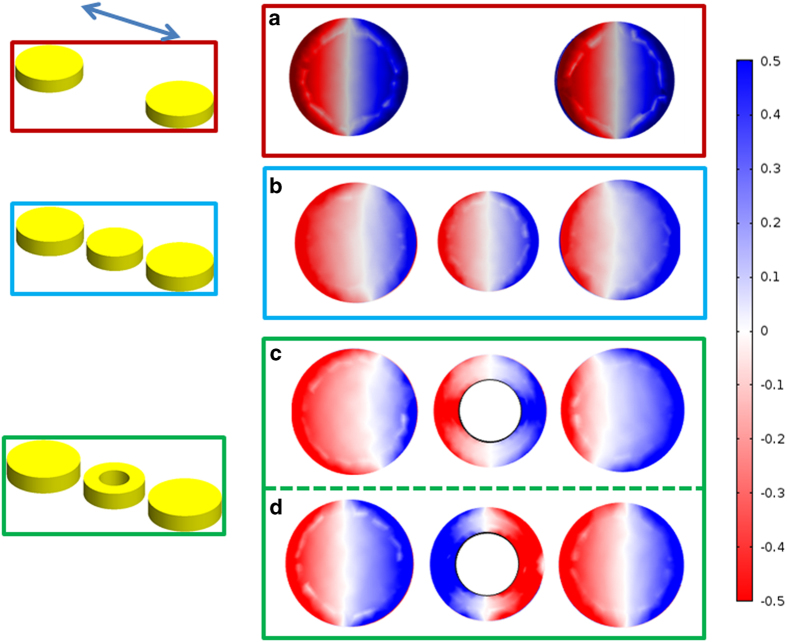
Charge distributions at the resonance wavelength for parallel polarization incidence. (**a**) No load computed at 632 nm. (**b**) Solid circular disk load computed at 705 nm. (**c**) and (**d**) Ring load computed at 845 nm and 622 nm, respectively.

In what follows we present the electromagnetic fields as well as the polarization charge distributions for the three nanoparticle configurations, shown in Figs. 6,7, under parallel excitation, computed at the resonance wavelengths. Note here that for the field quantities their normalized absolute value is displayed, while the imaginary part is plotted for the surface polarization. First, all the surface plots for the unloaded dimer are computed at 632 nm. It can be clearly seen that each particle exhibits the typical electric field distribution of a resonating dipole (high field intensity around the poles of the particle). Additionally, the magnetic field circulates around the two particles (in and out of the paper), while it exhibits its maximum along the particles’ equator. The field distribution is symmetric with respect to the symmetry plane of the particles indicating that there is minute coupling between them, and thus they radiate independently. However, since the two particles are identical their resonances occur at the same wavelength and thus the structure’s overall optical response exhibits a single resonance peak. The fact that the two particles function as two decoupled dipoles is further supported by the surface polarization charge shown in Fig. 7(a). It can be clearly seen that positive and negative charge is symmetrically distributed with respect to the particle’s equator. Note here that for a charge balanced dipole, the electric field vector is directed from positive to negative charge, while the magnetic field attains its maximum value along a plane perpendicular to the vector that connects the two charges.

For the second particle arrangement, which corresponds to the circular solid disk load, we first examine its polarization surface charge plots. For this loading scenario all surface plots are computed at 705 nm. Evidently, at resonance the induced charge on the three particles resembles that of three aligned dipoles. Now, from the distribution of the magnetic field intensity it is evident that a circulating magnetic field has been established around all three particles. In addition, the circulation of the magnetic field around each particle is synchronized so as to create the equivalent effect of a magnetic field circulating around an effective nanobar. As a matter of fact the strong electric field created between the middle and the outer particles affects the charge balance distribution on the latter. In particular, the charge is not evenly distributed on the outer particles and therefore, the magnetic field distribution around them is not symmetric with respect to their equator, but rather it squints towards the center particle. The two squinted magnetic field distributions, along with the center particle’s magnetic field distribution, create a combined effect that resembles the effective nanobar response mentioned previously. Consequently, due to the structure’s longer effective length it resonates at a red-shifted wavelength, longer than that of the individual particles.

For the nanoring loaded dimer, the field and charge distributions were computed at the two resonant wavelengths of the nanostructure, namely at 622 nm and at 845 nm. At the longer wavelength resonance of 845 nm, where the three particles function collectively, the field distribution closely resembles the one observed in the previously discussed case of the solid disc load. In particular, from Fig. 6(c) we can see that the strong electric field established within the structure’s gaps creates unbalanced charge distribution on the outer particles, as illustrated in Fig. 7(c). Consequently, similar to what was described previously, the magnetic field circulating around the outer particles squints towards the center particle, thus creating the effect of a magnetic field circulating around an effective nanobar. Obviously, due to the increased effective length of this nanobar, its resonance occurs at a red-shifted longer wavelength as shown in Fig. 4(i). It should be emphasized here that the coupling achieved in this case is considerably stronger than that created by the solid disc load and this is the reason why the resonance peak shifts to 845 nm, which is at a much longer wavelength than that corresponding to the solid disc loaded structure.

A totally different field and charge distribution is observed at the short wavelength resonance, at 622 nm. First, from Fig. 7(d) it is evident that the nanoring has opposite charge distribution with respect to that established on the outer particles. Because of this charge distribution the electric field vector around the center particle is directed oppositely with respect to the electric field vector established around the outer particles. Consequently, the sense of the magnetic field rotation around the center particle is also opposite to the rotation around the two outer particles. In addition to that, the magnetic field that circulates around the center particle is weaker than that corresponding to the outer particles. This is also evident from the electric field distribution shown in Fig. 6(d), where more electric field is concentrated around the outer particles rather than the center one. As a consequence, the electromagnetic response of the three particles decouples, and the trimer’s response is dominated by the contribution from the two outer particles. This is further justified by the short wavelength resonance of the structure which is very close to the one expected by the outer particles.

## Discussion

In conclusion, the feasibility of devising tunable dimer type nanoantennas has been demonstrated using plasmonic nanorings. Nanorings constitute a class of wavelength dependent nanoswitches whose electromagnetic properties (*e.g.* the two distinct dipole moments) can be tuned as desired by modifying their geometrical characteristics. It was shown that any modification of the nanoring geometry can be directly translated to an effective material property governed by an appropriate mixing rule. The latter is of paramount importance because it signifies that nanorings inherently exhibit tunable nanocircuit characteristics, and therefore it is expected that their applicability and utilization can be extended far beyond that of a two-state nanoswitch. For the nanoantenna configurations examined in this paper it was successfully demonstrated both numerically and most importantly experimentally, following a high accuracy fabrication process, that a dimer which is typically characterized by a single extinction resonance, can exhibit a dual mode operation. This was enabled by loading the dimer with a switching nanoring that permits its two component particles to either couple or decouple, and therefore to radiate either separately or collectively.

## Methods

### Numerical full wave simulations

All numerical simulations were performed using the COMSOL Multiphysics software package. For the simulations where the structure was excited with either a parallel or a vertical polarization the structure’s symmetry planes were exploited to reduce the computational load. For the 45° cases (unpolarized light) the entire structure was modeled.

### Device fabrication

Optimized ring-loaded and unloaded dimer nanoantennas were fabricated using a conventional top-down electron-beam lithography and metal lift-off process. First, a 150 nm thick electron-beam resist layer (Nippon Zeon ZEP 520A diluted by 50% with Anisole) was spun on a cleaned fused silica wafer at a speed of 5000 revolution per minutes (RPM) for 45 sec. The resist was soft-baked at 180 °C for 3 min, and the features were exposed at a dose of 190 μC/cm^2^ (Vistec EBPG 5200, spot size 7 nm and beam current 1 nA). The electron beam resist layer was developed at −10 °C in n-Amyl Acetate for 2 min and then MIBK: IPA = 8:1 for 1 min to remove n-Amyl Acetate. Cold development increased the contrast of the resist, which was required to reproducibly fabricate the ring-loaded nanoantenna structures with sub-10 nm control over the feature dimensions. After lithographic patterning, the nanoantenna structures were completed by electron-beam evaporating the Ti/Au (1 nm/30 nm) metal and lifting-off the deposited metal film by dissolving the resist in Microposit Remover 1165 (Rohm & Hass).

### Optical characterization

Unpolarized white light, generated by a tungsten-halogen lamp using an oil dark-field condenser with (NA = 1.2–1.43), was shined on the optical nanoantennas in the transmission mode. The scattering spectrum was collected using an upright microscope (Nikon TE 200U). The scattered light from the nanoantenna was collected with a 100 times magnifying lens. The spectrum of the scattered light was subsequently analyzed with an imaging spectrometer (Andor, Sharmock 303). The contribution from the substrate was removed by subtracting the spectra from adjacent unpatterned regions of equal size from the nanoantenna scattering spectra. The intensity variation of the light source was also accounted for by normalizing the corrected spectrum to the spectral intensity of the light source.

## Author Contributions

A.H.P. conceived the idea of using plasmonic nanorings to tune the scattering response of a dimer nanoantenna. A.H.P. designed the nanostructures, performed the numerical simulations and the effective medium analysis. Y.A.Y. fabricated the sample and performed optical characterization. Y.A.Y. and A.H.P. analyzed the data. D.H.W. and T.S.M. supervised the project. All authors co-wrote the manuscript.

## Additional Information

**How to cite this article**: Panaretos, A. H. *et al.* Tuning the optical response of a dimer nanoantenna using plasmonic nanoring loads. *Sci. Rep.* **5**, 9813; doi: 10.1038/srep09813 (2015).
