# Corrigendum: Tuning the optical response of a dimer nanoantenna using plasmonic nanoring loads

**DOI:** 10.1038/srep21942

**Published:** 2016-03-04

**Authors:** Anastasios H. Panaretos, Yu A. Yuwen, Douglas H. Werner, Theresa S. Mayer

Scientific Reports 5: Article number: 9813; 10.1038/srep09813 published online: 05112015; updated: 03042016.

The Acknowledgements section was omitted from the Article. It should read:

**Acknowledgements**

This work was supported by the Center for Nanoscale Science, an NSF Materials Research Science and Engineering Center, under the award DMR-1420620.

